# From Skull Density Ratio‐based eligibility to efficiency‐informed planning: Predictive modeling of thermal response in MR‐guided focused ultrasound

**DOI:** 10.1002/mp.70652

**Published:** 2026-09-10

**Authors:** José Angel Pineda‐Pardo, Jaime Caballero‐Insaurriaga, Marta Castillo‐Ortiz, Miguel López‐Aguirre, Rafael Rodríguez‐Rojas, Jorge U. Máñez‐Miró, Michele Matarazzo, Marta Del Alamo, Raul Martínez‐Fernández

**Affiliations:** ^1^ HM CINAC (Centro Integral de Neurociencias Abarca Campal) Hospital Universitario HM Puerta del Sur HM Hospitales Móstoles Madrid Spain; ^2^ Instituto de Investigación Sanitaria HM Hospitales (iHMH) Móstoles Madrid Spain; ^3^ Centro de Investigación Biomédica en Red de Enfermedades Neurodegenerativas (CIBERNED) Instituto de Salud Carlos III Madrid Spain; ^4^ PhD Program in Technologies for Health and Well‐being Polytechnic University of Valencia Valencia Spain; ^5^ Molecular Imaging Technologies Research Institute (I3M) Polytechnic University of Valencia Valencia Spain; ^6^ Centro Universitario HM Hospitales de Ciencias de la Salud (CUHMED) Universidad Camilo José Cela Madrid Spain

**Keywords:** acoustic energy, diploe thickness, MRgFUS, MR‐guided focused ultrasound, skull density ratio, skull thickness, temperature prediction, thermal efficiency

## Abstract

**Background:**

Transcranial MR‐guided focused ultrasound (MRgFUS) enables incisionless thermoablation of deep brain targets and has become an established modality in functional neurosurgery. Treatment efficiency is strongly influenced by skull‐mediated ultrasound attenuation. Although the skull density ratio (SDR) is currently the primary parameter used for pre‐procedural screening, it provides only a partial description of skull‐related variability in thermal response and offers limited guidance for intra‐procedural energy titration.

**Purpose:**

To comprehensively characterize skull‐related determinants of thermal efficiency beyond SDR and to develop multivariate models for predicting treatment efficiency and sonication‐wise peak temperature to support patient screening and intra‐procedural decision‐making.

**Methods:**

We retrospectively analyzed 316 MRgFUS thermoablative procedures (214 thalamotomies and 102 subthalamotomies). Skull metrics derived from CT included SDR, skull thickness (ST), diploe thickness (DT), angle of incidence (AOI), and higher‐order distributional descriptors. Thermal efficiency was quantified using multiple temperature‐energy‐based estimators. Two predictive models were developed, each validated on an independent held‐out test set (20% of procedures): (1) a forward‐selected multivariate linear regression model for procedure‐level thermal efficiency (temperature‐to‐energy ratio at 55°C‐TER_55_), guided by adjusted *R* ^2^ and AIC, with performance reported as *R* ^2^; and (2) a gradient boosting model for sonication‐wise peak temperature prediction, integrating sonication parameters, skull metrics, and cumulative treatment history, with performance reported as MAE globally and stratified by temperature range.

**Results:**

SDR, ST, and DT were significantly associated with thermal efficiency, and composite metrics such as SDR/DT outperformed SDR alone. A five‐feature multivariable linear regression model based on skull‐derived metrics achieved an adjusted *R* ^2^ of 0.63 (cross‐validated *R* ^2^ = 0.61, MAE = 0.54°C/kJ) for predicting TER_55_, with consistent generalization on an independent held‐out test set (*R* ^2^ = 0.58, MAE = 0.53°C/kJ). The peak temperature prediction model achieved a cross‐validated MAE of 1.70°C on the training set and 1.91°C on a fully held‐out procedure‐level test set, with stable performance across clinically dominant temperature ranges (< 60°C). Prediction accuracy was reduced at ≥60°C, reflecting data imbalance and systematic underestimation at extreme temperatures.

**Conclusions:**

Thermal efficiency in MRgFUS cannot be adequately characterized by SDR alone. A multivariate efficiency model integrating five skull descriptors substantially outperforms SDR‐based screening, providing a more informative and continuous representation of treatment feasibility and enabling pre‐procedural estimation of expected energy requirements. When combined with treatment parameters and cumulative thermal history, multivariate models enable accurate sonication‐wise temperature prediction, supporting efficiency‐informed energy titration and dynamic procedural guidance, and advancing MRgFUS toward more precise and personalized therapy.

## 1 INTRODUCTION

Transcranial MR‐guided focused ultrasound (MRgFUS) enables precise, incisionless therapeutic lesioning of deep brain structures.^1^, ^2^ This technology has revolutionized the field of functional neurosurgery, where essential tremor and Parkinson's disease have been major indications. MRgFUS thermoablation efficiency depends on skull‐mediated ultrasound attenuation, whereby skull properties impact thermal response to delivered acoustic energy (i.e., thermal efficiency). Currently, the skull density ratio (SDR), defined as the ratio between cortical and trabecular bone densities, is the primary pre‐procedure parameter for evaluating treatment feasibility.^3^, ^4^ Low SDR values (< 0.4) are associated with higher ultrasound attenuation and lower thermal efficiency, often requiring increased energy to reach ablative temperatures.^5^, ^6^, ^7^, ^8^ However, relying solely on SDR might be insufficient, as some low‐SDR cases may still be treatable.^8^, ^9^, ^10^ Others with moderate SDR can exhibit unexpectedly poor efficiency.^7^ While SDR provides general guidance, a more refined multivariate approach that integrates additional measures could improve prediction of temperature increase dynamics during treatment, particularly for cases with middle‐range SDR [0.3–0.5], where uncertainty is higher.^7^, ^11^, ^12^, ^13^

On the other hand, intraoperative decisions on sonication power and duration often rely on trial‐and‐error and operator expertise. While the SDR may guide initial sonication settings, there is no systematic approach to adjust parameters dynamically to ensure a controlled temperature increase. This lack of precision can lead to an excessive number of sonications or undesired temperature overshoots that increase both treatment time and the likelihood of adverse events. Consequently, developing accurate predictive models that link sonication parameters to expected temperature rise could significantly improve procedural efficiency, reduce reliance on real‐time adjustments, and enhance patient safety. Beyond ultrasound attenuation and energy delivery, additional factors may contribute to variability in thermal efficiency, including biological changes within the target region (e.g., edema and necrosis), which can alter ultrasound absorption and heat dissipation.^14^ Given these complexities, we propose incorporating not only sonication parameters (power, duration, and energy) and skull‐related attenuation features but also accumulated factors such as total delivered energy and cumulative thermal dose into these thermal rise predictive models.

Importantly, it is commonly assumed that skull‐based efficiency criteria can be directly transferred across targets. However, thermal efficiency is inherently target‐dependent. Changing the target modifies multiple interacting factors, including ultrasound incidence angles and SDR across elements. Consequently, identical skulls can exhibit markedly different thermal responses when treating different targets. To explicitly address this target dependency, we considered two clinical cohorts corresponding to thalamic and subthalamic thermoablation procedures, enabling both target‐specific analyses and a unified modeling framework.

Within this context, this study aims to address two key aspects of MRgFUS thermal efficiency: (1) developing a multiparametric predictive model that integrates skull‐derived properties beyond SDR, providing a more comprehensive framework for pre‐procedure screening; and (2) designing a predictive model for temperature rise, enabling precise, data‐driven adjustment of sonication parameters to optimize treatment execution.

## 2 METHODS

### 2.1 Participants and cohort definition

A retrospective cohort of 316 MRgFUS thermoablation procedures performed at HM‐CINAC between July 2015 and January 2024 was included in this study. Patients with essential tremor (ET) or Parkinson's disease (PD) who underwent MRgFUS thalamotomy ^15^, ^16^ or subthalamotomy ^17^, ^18^ at our institution (Hospital Universitario HM Puerta del Sur, Madrid, Spain) were screened. Of the included procedures, 214 corresponded to Vim‐FUS thalamotomy, primarily for ET (177 patients), but also for Parkinson's disease tremor (PDT, 35 patients) and multiple sclerosis‐associated tremor (MST, 2 patients).^19^ In addition, 102 procedures corresponded to STN‐FUS subthalamotomy for the treatment of the cardinal motor signs of PD.^17^ The Vim‐FUS group had a mean age of 69.5 years (SD = 10.7) and a sex distribution of 65/35% (M/F), whereas the STN‐FUS group had a mean age of 62.0 years (SD = 10.8) and a sex distribution of 71/29% (M/F). Among all procedures, 14 were bilateral (8 Vim‐FUS and 6 STN‐FUS), and 8 were retreatments (6 Vim‐FUS and 2 STN‐FUS) due to clinical relapse. All patients had an SDR above 0.3, which represents the minimum threshold for treatment at our institution. The study was approved by the Research Ethics Committee (CEIm) of HM Hospitales, Madrid, Spain (ref. 26.01.2653‐GHM). The study was conducted in accordance with the Declaration of Helsinki. Written informed consent was obtained from all patients prior to brain CT acquisition and treatment.

### 2.2 MRgFUS interventions

Thermoablative MRgFUS interventions were performed in an ExAblate 4000 system (InSightec, Haifa, Israel). CT images were acquired on a Toshiba Acquilion Prime scanner prior to the intervention using a bone enhancement convolution kernel. CT images were analyzed for patient screening using SDR cutoffs. Target location within the thalamic ventral intermediate (Vim) nucleus or the subthalamic nucleus (STN) was defined on the intra‐procedure MRI, as previously described.^15–^, ^18^ As a standard procedure, target‐to‐focus alignment was confirmed during treatment with low‐energy sonications under MR thermometry planes at the beginning of the procedure. Acoustic energy was then escalated, progressing from sub‐ablative and clinically reversible sonications to confirm therapeutic target engagement toward ablative sonications once clinical efficacy and safety were determined. In the absence of intra‐procedure complications, stopping criteria were sustained clinical improvement and delivery of an ablative thermal dose at the target, operationalized as either at least two sonications achieving peak average temperature ≥55°C or at least one sonication ≥60°C.

### 2.3 Skull data collection

**FIGURE 1:**
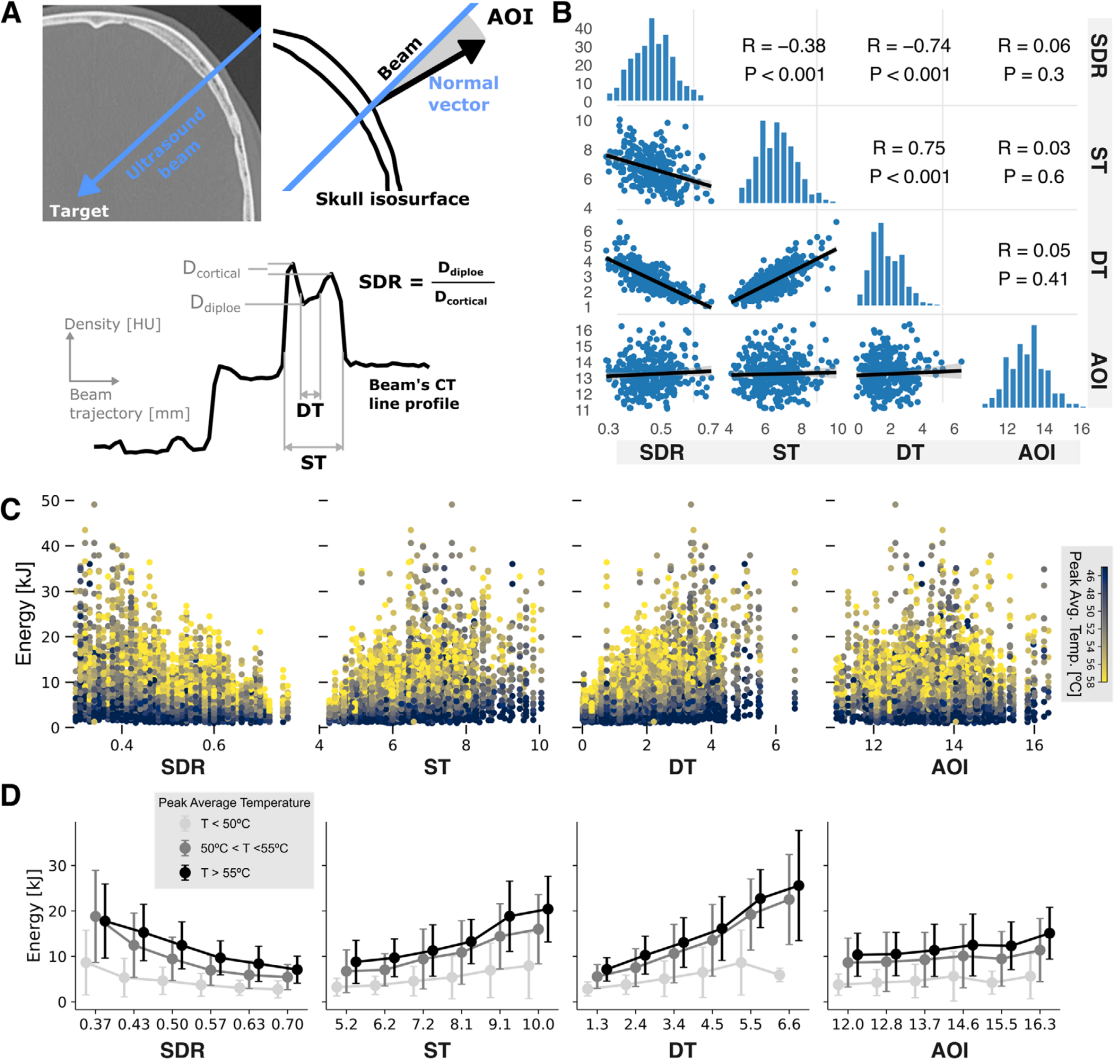
Skull parameters and their implications in energy‐temperature relationships. (a) Schematic representation of skull metrics, including skull density ratio (SDR), skull thickness (ST), and diploe thickness (DT) derived from CT images. The angle of incidence (AOI) is illustrated as the angle between the beam trajectory and the normal vector to the skull isosurface. (b) Pairwise correlation matrix between skull average metrics, showing Pearson correlation coefficients and significance levels. (c) Scatter plots showing individual sonications, where skull metrics (SDR, ST, DT, AOI) are plotted against delivered energy, with peak average temperature achieved represented in color (gray to yellow). (d) Error‐bar plots depicting the relationship between skull metrics and delivered energy. Sonications are now grouped into three thermal ranges: [<50°C] (light gray), [50–55°C] (dark gray), and > 55°C (black). Average values and standard deviations are shown.

Treatment reports were retrospectively obtained containing a summary of skull parameters seen by each ultrasound element for target coordinates. These skull parameters were estimated using proprietary Insightec algorithms based on CT intensity profiles sampled along straight‐line beam paths from each ultrasound element to the target. The SDR for each ultrasound element was estimated as the ratio of CT intensities between the diploe and the cortical bone and computed as the local minimum between the two peaks (diploe) divided by the mean of the two maxima (cortical bone).^11^ These values were averaged to obtain a single SDR estimate reflecting the overall skull bone homogeneity for each subject. In addition, skull thickness (ST) and diploe thickness (DT) were extracted, defined as the distance between the entry and exit points of the ultrasound beam across the skull and the diploe, respectively. The angle of incidence (AOI) was computed as the angle between the ultrasound beam trajectory and the normal vector to the skull surface (Figure 1a). All skull metrics (SDR, ST, DT, and AOI) were subjected to robust outlier filtering prior to aggregation. Outliers were identified using a *z*‐score threshold greater than 3.5. Elements with non‐physiological values (e.g., DT = 0), likely arising from segmentation or ray‐tracing artifacts, were excluded. Filtered element‐wise values were then averaged to obtain subject‐level estimates. AOI values were converted to degrees for all subsequent analyses. To further account for the interaction between skull density and thickness, the ratios SDR/ST and SDR/DT were computed element‐wise across transducer elements and subsequently averaged. These ratios were scaled by a factor of 10 for numerical convenience. Finally, motivated by evidence that mean values alone may insufficiently capture thermal efficiency,^20^ higher‐order distributional descriptors, standard deviation, skewness, and kurtosis, were computed for all skull‐related metrics to characterize inter‐element heterogeneity. Available skull area (mm^2^) and number of active elements (NoEl) were also collected.

### 2.4 Treatment data collection and thermal efficiency

For each sonication, duration, acoustic power, delivered energy, and peak average temperature at the target were recorded. Procedural metrics also included thermal efficiency (estimated as ∆T/Energy),^21^ and thermal dose in cumulative equivalent minutes at 43°C (CEM43).^22^ Temperature evolution along each sonication was approximated as a linear increase from baseline body temperature (37°C) to the measured peak average temperature over the duration of the sonication, followed by a linear cooling phase returning to 37°C over a period equal to 1.5 times the sonication duration. Thermal dose values were log‐transformed as log_10_(1+CEM43). To capture history‐dependent effects on heat transfer, accumulated acoustic energy, and accumulated thermal dose up to the preceding sonication were computed (E_acc(n‐1)_ and CEM43_acc(n‐1)_, respectively). These cumulative measures were included as predictors of model potential carry‐over effects on thermal response during subsequent sonications. Further, summary treatment metrics included the total number of sonications (NoS), average energy per sonication (E_avg_), accumulated delivered energy (E_accu_), maximum achieved temperature (T_max_), and the corresponding delivered energy at T_max_ (E_max_).

Treatment thermal efficiency was quantified from whole‐treatment temperature‐energy profiles derived from individual sonications (see Figure S‐1). Three complementary efficiency metrics were also computed. First, the temperature‐to‐energy ratio at the maximum achieved temperature (T_max_), denoted as TER_max_, was defined as the ratio between the temperature rise at T_max_ (Δ T_max_ = T_max_‐37ºC) and the corresponding delivered energy.^3^ Second, the slope of the temperature‐energy relationship obtained from a logarithmic fit T=A·ln(E)+B, where the multiplicative coefficient A was used as the measure of temperature‐to‐energy steepness (TES), reflecting the sensitivity of temperature rise to incremental energy delivery.^11^, ^23^ Third, the temperature‐to‐energy ratio at 55°C (TER_55_), estimated by computing the energy required to reach 55°C (E_55_) from the logarithmic fit and defining TER_55_ = ∆T/E_55_.^24^ Analogous TER_xx_ and E_xx_ metrics were also derived for target temperatures of 45°C, 50°C and 60°C.

### 2.5 Statistics

Continuous variables were reported as mean and standard deviations. To investigate target‐dependent effects, skull metrics, treatment performance variables, and thermal efficiency measures were systematically compared between the Vim‐FUS and STN‐FUS groups. Between‐group differences were assessed using unpaired two‐sample *t*‐tests. Associations between continuous variables were evaluated using Pearson's linear correlation. Linear regression analyses were performed to assess the ability of skull‐related metrics to explain variability in thermal efficiency, with coefficients of determination (*R* ^2^) reported. Statistical significance was set at *P* < 0.05. All statistical analyses were conducted using R (v4.0.2) and Python (v3.11.6).

To identify an optimal subset of predictors for treatment thermal efficiency (TER_55_), a forward feature selection procedure was implemented using multiple linear regression. Each procedure, including bilateral treatments and retreatments in the same biological patient, was treated as an independent observation, as each involves a different transducer‐to‐skull geometry determined by stereotactic frame positioning or target coordinates (i.e., bilateral treatments), resulting in distinct acoustic trajectories and skull segments traversed by the ultrasound beam. Procedures passing quality control (logistic fit *R* ^2^ ≥ 0.75, fitting MAE ≤ 7.5°C) with complete skull‐derived metrics were divided into a training set (80%) and a held‐out test set (20%) stratified by TER_55_ quintiles. Feature selection was guided by adjusted *R* ^2^ and the Akaike Information Criterion (AIC), starting from an intercept‐only model and sequentially evaluating candidate predictors. At each step, a candidate was retained only if it improved model fit while all selected variables maintained VIF ≤ 5. Internal validation employed 5‐fold cross‐validation with procedure‐level grouping on the training set, and final generalization was assessed on the held‐out test set using the selected multiple linear regression model. An efficiency‐weighted variant (sample weights ∝ $1/TE{R_{55}}^{0.8}$) was additionally evaluated to improve prediction accuracy for low‐efficiency cases.

For peak temperature prediction, a sonication‐wise regression framework was adopted. Sonications were retained only if their energy‐temperature profile met quality‐control thresholds (logistic fit *R* ^2^ ≥ 0.75, fitting MAE ≤ 7.5°C). The resulting dataset was then divided into a training set (80%) and a held‐out test set (20%) using stratified random sampling by temperature bin, ensuring that all sonications from a given procedure remained within the same partition to prevent data leakage. We first considered a core set of sonication‐level variables (acoustic power, duration, and delivered energy) and evaluated the contribution of additional skull and treatment descriptors through forward feature selection. At each step, the candidate minimizing the 10‐fold cross‐validated MAE was added, provided it reduced MAE beyond a minimum improvement threshold (0.005°C) and did not introduce multicollinearity (VIF ≤ 8), a threshold consistent with the use of tree‐based ensemble models as the final prediction framework. The AIC was used as a secondary stopping rule to penalize model complexity. Candidate variables included skull morphometry metrics previously identified as relevant for thermal efficiency, as well as cumulative energy E_acc(n−1)_ and thermal dose CEM43_acc(n−1)_. Feature selection was performed exclusively on the training set, i.e. no test‐set information was used at any stage. In addition, a minimal set combining only the core sonication parameters with SDR, skull area and the number of active elements (minimal set) was evaluated as a pragmatic clinical baseline.

Both feature sets (optimal and minimal) were used to train gradient boosting regression models (150 trees, learning rate 0.10, maximum depth 3, column subsampling 0.85, L2 regularization 1.0, minimum samples per leaf 10). Models were fitted on complete cases, with sonications lacking any required covariate excluded independently for each feature set. Hyperparameters were fixed a priori to favor conservative regularization and were not optimized via automated search. Internal validation employed stratified 10‐fold cross‐validation at the sonication level, with fold assignments stratified by peak temperature bins (< 50, 50–55, 55–60, ≥60°C) to ensure balanced representation across the temperature range. Given the underrepresentation of high‐temperature sonications (≥60°C, 3.3% of training data), an oversampling variant was additionally evaluated in which ≥60°C sonications were replicated three times within each training fold. Final model performance was reported on the held‐out test set using MAE, both globally and stratified by temperature range. To assess the relative contribution of each predictor, permutation feature importance was computed on the training set by measuring the increase in MAE after randomly shuffling each variable independently (20 repetitions per feature).

## 3 RESULTS

### 3.1 Treatment summary details

Treatment parameters differed between Vim‐FUS and STN‐FUS groups. The STN‐FUS cohort showed differences in skull‐related parameters, with a larger skull area and fewer active elements compared with Vim‐FUS. STN‐FUS also received a higher number of delivered sonications overall, with a greater number of therapeutic sonications exceeding either low‐ or high‐temperature thresholds (> 50°C and > 55°C). In addition, STN‐FUS exhibited higher average power, as well as higher average, maximum, and accumulated energy, while the average sonication duration was comparable between groups. Maximum peak temperature was slightly higher in the STN‐FUS group (Table 1).

**TABLE 1:** Treatment summary for Vim‐FUS and STN‐FUS groups.

	Vim‐FUS (*N* = 214)	STN‐FUS (*N* = 102)	*P*‐value
Skull area (cm^2^)	356.0 (31.9)	367.8 (37.1)	**0.0042**
No. active elements	946 (36)	914 (58)	**< 0.001**
No. delivered sonications	14 (5)	17 (5)	**< 0.001**
No. delivered sonications > 50°C	10 (4)	14 (4)	**< 0.001**
No. delivered sonications > 55°C	5 (2)	7 (3)	**< 0.001**
Average sonication power (W)	778 (207)	876 (196)	**< 0.001**
Average sonication duration (s)	11.1 (2.8)	11.5 (2.6)	n.s.
Average sonication energy (kJ)	8.2 (4.0)	9.5 (3.7)	**0.0091**
Maximum sonication energy (kJ)	15.4 (8.3)	17.3 (7.0)	**0.0471**
Accumulated sonication energy (kJ)	111.8 (64.6)	155.7 (61.4)	**< 0.001**
Peak average temperature (°C)	59.6 (2.5)	60.3 (3.1)	**0.0342**

*Note*: Reported parameters include skull area, number of active elements, total delivered sonications, and counts of low‐temperature and high‐temperature therapeutic sonications (> 50°C and > 55°C), average power, average duration, average/maximum energy, accumulated energy, and peak average temperature across sonications. Values are reported as mean (standard deviation). Group comparisons were performed using unpaired two‐sample *t*‐tests, with their corresponding *P*‐values reported.

### 3.2 Skull properties and energy‐temperature relationship

Average skull metrics across the whole cohort were: SDR = 0.54 ± 0.11, ST = 6.61 ± 1.11 mm, DT = 2.71 ± 0.92 mm, and AOI = 13.12 ± 1.05°. No significant differences were observed between the Vim‐FUS and STN‐FUS groups for any of these parameters, or for derived ratios (SDR/ST and SDR/DT). While mean AOI was similar between groups (Vim: 13.13° ± 0.96°; STN: 13.09° ± 1.21°), the standard deviation of AOI was significantly larger in STN‐FUS (6.23° ± 0.80° vs. 5.88° ± 0.79°; *P *< 0.001). This greater AOI dispersion was associated with fewer active elements (*R* = ‐0.29, *P *< 0.001). In addition to AOI dispersion, several skewness‐related metrics differed between groups, including ST skewness (*P* = 0.015), DT skewness (*P* = 0.033), AOI skewness (*P* = 0.017), and SDR/ST skewness (*P *= 0.004). No significant group differences were observed in kurtosis measures for any skull parameter. A comprehensive summary of all skull metrics and statistical comparisons is provided in Table S‐1.

SDR, ST, and DT were significantly correlated between each other (Figure 1b), while AOI was dissociated from them. ST and DT were highly correlated (*R* = 0.75, *P* < 0.001). Moreover, SDR displayed a negative correlation with both ST (*R* = −0.38, *P *< 0.001), and DT (*R* = −0.74, *P *< 0.001), that is, higher SDR scores were related to smaller thickness in skull and diploe. The required energy to achieve a specific thermal rise in the target was associated with SDR, ST, and DT (Figure 1c‐d). The slope of this relationship indicates the strength of the influence of a skull property in the heating capacity, and was more pronounced for higher peak temperatures on target (above 55°C). This slope was also steeper for DT than for the SDR or ST, suggesting a higher contribution of this metric to thermal efficiency loss due to skull attenuation.

### 3.3 Treatment efficiency and skull properties

**FIGURE 2:**
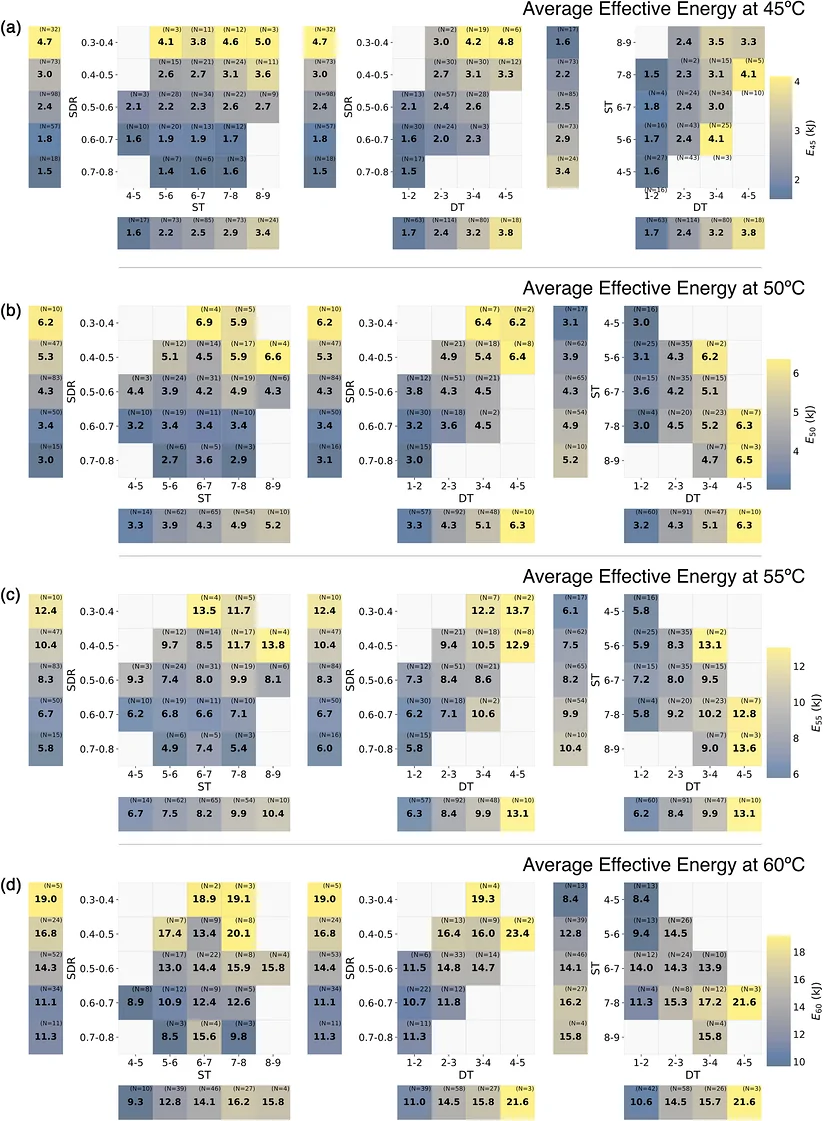
Heat maps showing the average energy required to reach target temperatures of 45°C (a), 50°C (b), 55°C (c), and 60°C (d) as a function of skull parameters (SDR, ST, and DT). Energy values correspond to estimates obtained after logarithmic fitting of the energy‐temperature curves. Two‐dimensional heat maps represent conditional averages for each parameter combination, while one‐dimensional marginal heat maps show global averages per parameter bin, independent of the paired parameter. Only sonications with mean duration ≤ 12 s that met the corresponding temperature threshold were included. Cell values indicate mean energy (kJ), with sample size (*N*) shown in parentheses; cells with *N* < 2 were excluded from visualization.

A summary of thermal efficiency treatment descriptors is provided in Table 2. Across the whole cohort, mean values were TER_max_ = 2.00 ± 0.98°C/kJ, TER_55_ = 2.16 ± 1.04°C/kJ, and TES = 7.29 ± 1.69°C/ln(kJ). Despite minimal differences in average skull properties between treatment groups, the STN‐FUS group consistently exhibited lower thermal efficiency for several key metrics. Specifically, TER_55_, TER_60_, TES, and TERmax were significantly reduced in STN‐FUS compared with Vim‐FUS (*P *< 0.05), indicating that greater acoustic energy was required to achieve equivalent temperature increments in STN‐FUS procedures. There were no significant group differences for estimated energy to reach 50°C and 55°C, whereas E_60_ was significantly higher in the STN‐FUS group (*P *= 0.0403), further indicating reduced efficiency at higher target temperatures for STN treatments. In Figure 2a‐d, conditional heat maps illustrate the average efficiency or energy required to reach clinically relevant target temperatures (45, 50, 55, and 60°C) across combinations of skull parameters. These heat maps are restricted to parameter intervals with *N* > 2 observations, providing robust visualization of how skull composition modulates treatment efficiency within the studied cohort. Notably, substantial variability in thermal efficiency was observed even within narrow SDR ranges. For instance, within the low‐SDR range of 0.30–0.40, TER_55_ spanned 0.41‐1.99°C/kJ and E_55_ ranged from 9.1 to 43.7 kJ. Among the 7 procedures in our dataset in which the target temperature of 55°C was not reached, skull parameters spanned SDR 0.31–0.42, DT 3.26–5.53 mm, and ST 6.42–9.63 mm, ranges within which 12%, 25%, and 54% of successfully treated patients also fell, respectively. This overlap underscores that no single skull metric can reliably define exclusion criteria in isolation, supporting the rationale for multivariate predictive approaches as explored in the subsequent sections.

**TABLE 2:** Thermal efficiency metrics.

Efficiency Metric	All patients	Vim‐FUS	STN‐FUS	*P*‐value
TER_50_ (°C/kJ)	2.98 (1.29)	3.08 (1.37)	2.86 (1.06)	n.s.
TER_55_ (°C/kJ)	2.16 (1.04)	2.28 (1.11)	1.97 (0.82)	**0.0145**
TER_60_ (°C/kJ)	1.82 (0.81)	1.98 (0.90)	1.57 (0.52)	**0.0017**
TES (°C/ln(kJ))	7.29 (1.69)	7.48 (1.77)	7.00 (1.42)	**0.0174**
TER_max_ (°C/kJ)	2.00 (0.98)	2.12 (1.03)	1.81 (0.85)	**0.0096**
E_50_ (kJ)	5.98 (9.90)	6.08 (11.87)	5.37 (2.76)	n.s.
E_55_ (kJ)	11.31 (8.51)	10.52 (6.96)	11.44 (6.86)	n.s.
E_60_ (kJ)	15.34 (8.19)	14.26 (7.05)	17.02 (9.63)	**0.0403**

*Note*: Metrics are expressed in either temperature‐to‐energy ratio (°C/kJ) or absolute energy (kJ), summarizing the efficiency of energy delivery to achieve specific temperature thresholds. Values are reported as mean (SD) for all patients and separately for the Vim‐FUS and STN‐FUS groups. Group differences were assessed using two‐sample unpaired *t*‐tests. TER_T_, temperature‐to‐energy ratio at temperature T°C (°C/kJ). E_T_, acoustic energy required to reach T°C (kJ); TER_max_, temperature‐to‐energy ratio at maximum peak average temperature; TES, slope of the logarithmic temperature‐energy relationship (°C per unit increase in ln E).

**FIGURE 3:**
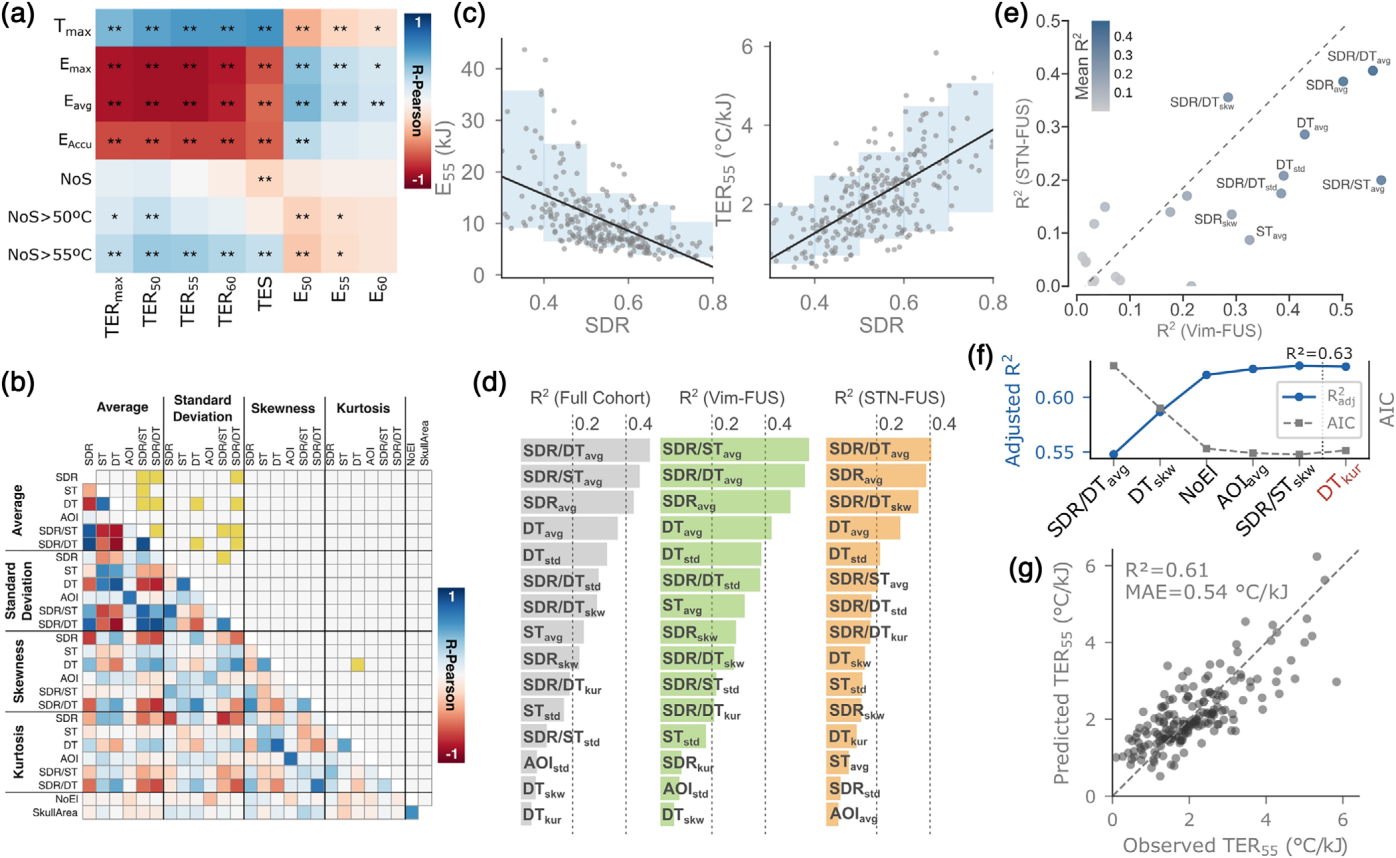
Temperature‐energy relationships and skull‐related predictors of thermal efficiency. (a) Inter‐subject correlation matrix between efficiency estimators and treatment summary metrics, including maximum temperature (T_max_), maximum energy (E_max_), average and accumulated energy (E_avg_, E_accu_), total number of sonications (NoS), and the number of sonications exceeding 50°C and 55°C. Colors indicate Pearson correlation coefficients (blue: positive; red: negative), with statistical significance denoted as *P < 0.05 and **P < 0.01. (b) Correlation matrix of skull‐derived metrics and their statistical estimators (mean, standard deviation, skewness, and kurtosis) for SDR, ST, DT, AOI, and the ratios SDR/ST and SDR/DT. (c) Scatterplots of SDR against E_55_ (left) and TER_55_ (right). Solid line: linear regression fit. Shaded rectangles indicate the 5th–95th percentile range within consecutive SDR bins (width = 0.10), illustrating the inter‐patient variability that persists across the SDR spectrum. (d) Bar plots showing the coefficient of determination (*R* ^2^) from univariate linear regressions between TER_55_ and individual skull parameters for the full cohort, Vim‐FUS, and STN‐FUS groups, respectively. Variables are ranked by decreasing explanatory power within each group. (e) Comparison of *R* ^2^ values for each skull parameter between the Vim‐FUS and STN‐FUS groups. The dashed diagonal indicates equal explanatory power across targets, highlighting parameters with group‐specific relevance. (f) Forward feature selection results for multivariable modeling of TER_55_, showing the evolution of adjusted *R* ^2^ (left axis) and AIC (right axis) as skull‐derived predictors are sequentially added. The vertical dotted line indicates the optimal model (5 features, adjusted *R* ^2^ = 0.63), beyond which additional variables do not improve the AIC criterion. Variables beyond the optimal set are shown in red. (g) Scatter plot of observed versus predicted TER_55_ from 5‐fold cross‐validation (*N* = 178 procedures). Each point represents an out‐of‐fold prediction. The dashed diagonal indicates perfect agreement (identity *y* = *x*). Cross‐validated *R* ^2^ = 0.61, MAE = 0.54°C/kJ.

These efficiency measures (TER_max_, TER_55_, and TES) demonstrated significant correlations with treatment summary metrics (Figure 3a), show negative correlations with E_max_, E_avg_, and E_accu_, and a positive correlation with T_max_, as expected. TES correlated negatively with the total NoS and positively with the NoS exceeding 55°C. TER_55_ did not show a significant correlation with the total NoS but was strongly associated with the number of sonications above 55°C. TER_max_ demonstrated a moderate correlation with NoS > 55°C. Furthermore, Figure 3b reveals strong collinearity among mean and standard deviation descriptors of SDR, ST, DT and their ratios, while AOI metrics remained largely independent. Skewness and kurtosis of SDR and SDR/DT correlated mainly with their corresponding mean values, informing variable selection for multivariable modeling.

Rather than modeling absolute energy values directly, we focused on predicting the efficiency‐based descriptors, which better reflected the underlying physical relationship between skull properties and thermal response. Since energy requirements scaled inversely with treatment efficiency, expressing efficiency as a temperature‐to‐energy ratio effectively linearized this relationship and yielded more stable and interpretable models (Figure 3c). Regarding efficiency metrics, TER_55_ was identified as a particularly useful estimator, as it accounts for the entire treatment procedure after a logarithmic fitting, unlike TER_max_, which was based on a single reading and may not accurately represent the entire process. Additionally, TER_55_ was easily translatable to therapeutic contexts, as it provided insight into the energy required to surpass the ablative threshold of 55°C.^25^ Conversely, while TES is unbiased, it did not correlate as strongly with treatment summary metrics, making it a less precise representation of treatment development.

The *R* ^2^ values for the univariate regression of TER_55_ against skull property metrics, SDR, ST, DT, AOI, and the ratios SDR/ST and SDR/DT, together with their statistical estimators are shown in Figure 3d. In the full cohort, the highest predictive performance was obtained for SDR/DT_avg_ (*R* ^2^ = 0.50), followed by SDR/ST_avg_ (*R* ^2^ = 0.46), SDR_avg_ (*R* ^2^ = 0.44), and DT_avg_ (*R* ^2^ = 0.37). Secondary contributions arose from dispersion and shape descriptors, including DT_std_ (*R* ^2^ = 0.33), SDR/DT_std_ (*R* ^2^ = 0.30), and SDR/DT_skw_ (*R* ^2^ = 0.29), whereas AOI‐derived metrics and skull area exhibited negligible explanatory power. Figure 3d also shows the same analysis stratified by target. In the Vim‐FUS subgroup, predictive performance increased globally, with SDR/ST_avg_ (*R* ^2^ = 0.57) and SDR/DT_avg_ (*R* ^2^ = 0.56) emerging as the strongest predictors, followed by SDR_avg_ (*R* ^2^ = 0.50) and DT_avg_ (*R* ^2^ = 0.43). DT_std_ and SDR/DT_std_ (*R* ^2^ = 0.39; *R* ^2^ = 0.38 respectively) showed moderate relevance. In contrast, the STN‐FUS subgroup exhibited lower overall prediction accuracies, with maximum *R* ^2^ values observed for SDR/DT_avg_ (*R* ^2^ = 0.41) and SDR_avg_ (*R* ^2^ = 0.39), followed by SDR/DT_skw_ (*R* ^2^ = 0.36) and DT_avg_ (*R* ^2^ = 0.29). Notably, SDR/ST_avg_ contributed less in STN‐FUS (*R* ^2^ = 0.20) compared with Vim‐FUS. Overall, SDR/DT_avg_, SDR_avg_, and DT_avg_ provided robust predictive information across both targets, while SDR/ST_avg_ was most informative for Vim‐FUS, and SDR/DT_skw_ gained relative importance in STN‐FUS (Figure 3e). A forward selection approach was applied using adjusted *R* ^2^ and AIC to optimize the predictive model for TER_55_ (Figure 3f). The selected features were SDR/DT_avg_, DT_skw_, NoEl, AOI_avg_, and SDR/ST_skw_, which achieved an adjusted *R* ^2^ of 0.63 (cross‐validated *R* ^2^ = 0.61, MAE = 0.54°C/kJ; Figure 3g). On the held‐out test set (40 procedures), the model yielded *R* ^2^ = 0.58 and MAE = 0.53°C/kJ for TER_55_, corresponding to a derived E_55_ prediction accuracy of *R* ^2^ = 0.35 and MAE = 4.1 kJ. An efficiency‐weighted variant, designed to improve accuracy for low‐efficiency procedures, achieved improved E_55_ prediction on the test set (*R* ^2^ = 0.48, MAE = 3.8 kJ) at the cost of a modest reduction in TER_55_ accuracy (*R* ^2^ = 0.60, MAE = 0.52°C/kJ). A cross‐validated comparison of SDR‐only, optimal, and efficiency‐weighted models is provided in Figure S‐2.

### 3.4 Factors that influence thermal efficiency

**FIGURE 4:**
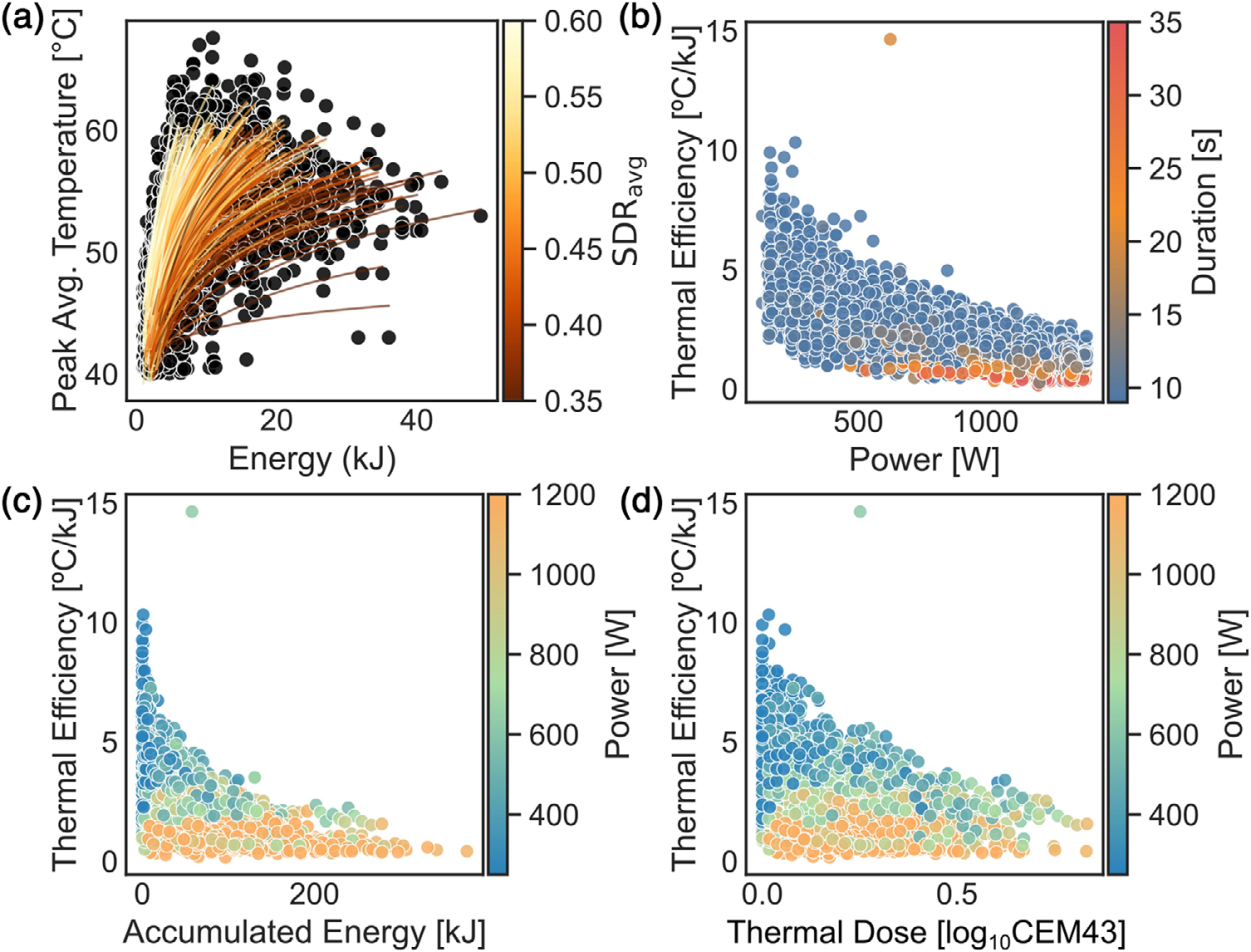
Nonlinear dependencies between acoustic delivery parameters and thermal efficiency. (a) Peak average temperature as a function of delivered acoustic energy, illustrating a saturating relationship in which temperature increments progressively diminish at higher energy levels. Line color encodes skull density ratio (SDR). (b) Thermal efficiency, defined as temperature increase per unit energy, plotted against applied acoustic power. Higher powers are associated with reduced efficiency, indicating non‐proportional heat deposition. (c) Relationship between accumulated acoustic energy and thermal efficiency, showing a monotonic decrease in efficiency with increasing cumulative energy, potentially reflecting time‐dependent skull or tissue effects. (d) Thermal efficiency as a function of thermal dose (log10CEM43), highlighting reduced efficiency at higher doses, which suggest additional non‐linear and tissue‐related contributions to heating dynamics.

The relationship between acoustic energy and peak temperature was non‐linear, with diminishing temperature gains at higher energy levels (Figure 4a). Accordingly, thermal efficiency decreased as delivered energy increased (Figure 4b). An inverse relationship was observed between thermal efficiency and cumulative acoustic energy (Figure 4c), indicating a progressive reduction in temperature gain per unit of energy across successive sonications. Similarly, thermal efficiency decreased with increasing cumulative thermal dose (Figure 4d). These findings indicate that both accumulated acoustic energy and cumulative thermal dose are associated with a progressive reduction in thermal efficiency during treatment.

### 3.5 Multivariate model of peak temperature prediction

**FIGURE 5:**
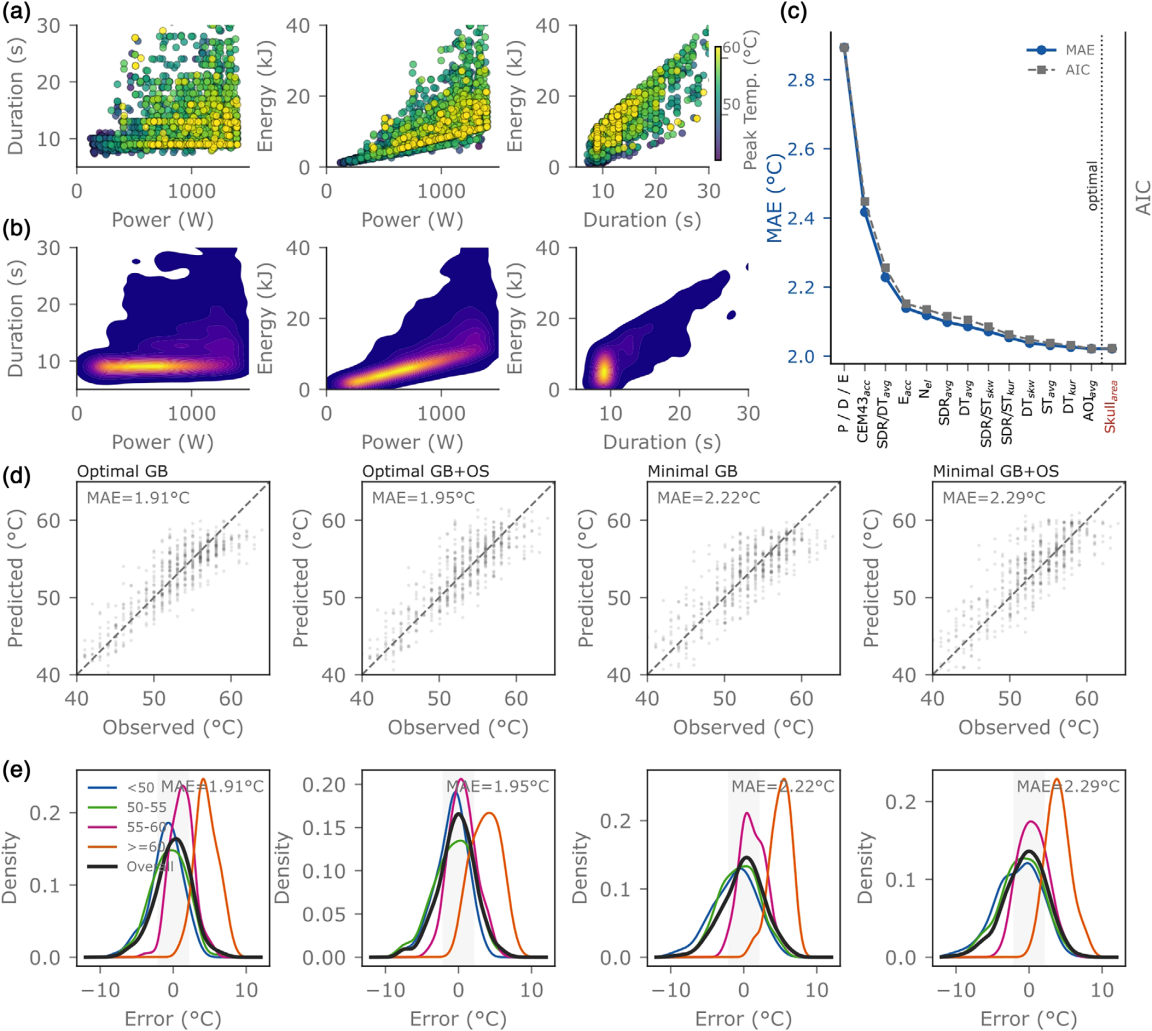
Multivariate modeling of peak temperature prediction. (a) Distribution of individual sonications in the power‐duration‐energy space, color‐coded by the achieved peak average temperature. (b) Bivariate two‐dimensional density estimates of the same physical relationships, highlighting the most frequent regions of the parameter space. (c) Evolution of the mean absolute error (MAE) as a function of the feature set; the dashed line indicates the baseline reference, and feature sets with the best accuracy‐complexity trade‐off are highlighted in red. (d) Scatter plots showing the relationship between true and predicted peak average temperature for the different models: minimal feature set, minimal set with oversampling, optimal feature set, and optimal set with oversampling; the dashed line represents identity (*y* = *x*). (e) Prediction error density distributions (True—Predicted) for each model, shown by temperature range, and overall, the gray shaded area denotes the ± 2°C error interval.

We trained and validated a sonication‐wise machine learning model to predict peak average temperature using 4138 sonications from 316 MRgFUS procedures. After quality control based on logarithmic fitting of energy‐temperature curves (*R* ^2^ ≥ 0.75 and fitting MAE ≤ 7.5°C) 3199 sonications from 238 procedures were retained. These were split into training (190 procedures, 2567 sonications) and held‐out test (48 procedures, 632 sonications) sets ensuring that no sonications from the same procedure appeared in both sets. The resulting dataset exhibited a marked temperature imbalance: 39.0% of sonications fell in the 50–55°C range, 26.4% in 55–60°C, 31.4% below 50°C, and only 3.2% above 60°C, which intrinsically limits predictive accuracy in the highest temperature regime (see Figure S‐3). Figure 5a–b illustrate the empirical operating space covered by the dataset in terms of power, duration, and energy, as well as their joint distributions, which defines the region in which model generalization is primarily supported. Model development therefore focused on capturing relationships within this observed parameter space. We first considered a core set of sonication‐level variables (power, duration, and energy) and evaluated the contribution of additional skull and treatment descriptors through forward feature selection. Candidate variables included skull‐related metrics (Figure 3f), as well as E_acc(n‐1)_ and CEM43_acc(n‐1)_.

Two Gradient Boosting feature configurations were evaluated. The first was a minimal operator‐accessible model, extending the core sonication parameters (Power, Duration, and Energy) with SDR_avg_, skull area, and number of active elements, all of which are readily available during clinical MRgFUS procedures and can be easily integrated into current workflows. The second was an “optimal” multivariable model, which, beyond Power, Duration, and Energy, incorporated only those skull‐related and history‐dependent covariates retained by the feature selection process (Figure 5c).

Performance was evaluated on the held‐out test set (48 procedures, 550 and 525 sonications for the optimal and minimal feature sets, respectively), which was not used during feature selection or model training. Results are reported both globally and stratified by peak temperature range (Figure 5d–e), with MAE and the proportion of predictions within predefined absolute error thresholds.

The optimal feature set comprised 15 variables: the three base sonication parameters (power, energy, duration), CEM43_acc(n‐1)_, E_acc(n‐1)_, NoEl, and nine skull‐derived metrics (SDR_avg_, DT_avg_, ST_avg_, AOI_avg_, SDR/DT_avg_, and higher‐order statistics of DT and SDR/ST distributions). Permutation importance analysis (see Figure S‐4) indicated that sonication energy, CEM43_acc_, and E_acc_ were the dominant predictors, followed by SDR_avg_ and SDR/DT_avg_, confirming that both treatment‐level and skull‐level covariates contribute meaningfully to prediction accuracy. The optimal model achieved an overall test MAE of 1.91°C, with 60.2% of sonications predicted within |error| ≤ 2°C and 81.5% within ≤ 3°C. Accuracy was consistent across the most prevalent clinical temperature regimes: MAE of 1.80°C (< 50°C), 1.97°C (50–55°C), and 1.58°C (55–60°C). In contrast, prediction accuracy degraded markedly for sonications ≥ 60°C (MAE 4.58°C), reflecting both strong class imbalance and increased thermometry variability at high temperatures. Applying training‐fold oversampling for ≥ 60°C sonications improved performance in this regime (MAE 3.77°C) while preserving comparable global accuracy (overall MAE 1.95°C, 62.7% within |error| ≤ 2°C). The minimal feature set showed reduced but still meaningful performance: overall test MAE of 2.22°C, with 53.7% within |error| ≤ 2°C. Errors were 2.48°C (< 50°C), 2.16°C (50–55°C), and 1.69°C (55–60°C), increasing substantially for ≥ 60°C (MAE 5.00°C). With oversampling, ≥ 60°C MAE improved to 4.01°C at a modest cost in overall error (MAE 2.29°C). Internal cross‐validation on the training set yielded consistent results, with a 10‐fold CV MAE of 1.70°C for the optimal set and 1.88°C for the minimal set (see Table S‐2). Across all configurations, accuracy was consistently higher below 60°C, underscoring the combined impact of data imbalance and intrinsic variability at higher temperatures. See Table 3 for a summary of all configurations. Calibration plots and 95% prediction intervals stratified by temperature bin are provided in Figure S‐5.

**TABLE 3:** Held‐out test set performance for peak temperature prediction (inclusion criteria: Logistic fit *R* ^2^ ≥ 0.75, fitting MAE ≤ 7.5°C).

Feature set	Training mode	Temp. range (°C)	*n*	MAE (°C)	≤3°C (%)	≤2°C (%)	≤1°C (%)
Optimal	No weight	<50	156	1.80	84.60	64.70	34.60
		50–55	216	1.97	79.60	57.40	29.60
		55–60	157	1.58	89.80	66.90	38.20
		≥60	21	4.58	14.30	4.80	0.00
		**Overall**	**550**	**1.91**	**81.50**	**60.20**	**32.40**
Optimal	Oversample ≥60°C	<50	156	1.79	81.40	67.30	38.50
		50–55	216	2.20	71.80	55.60	27.30
		55–60	157	1.51	87.30	73.20	40.80
		≥60	21	3.77	28.60	23.80	9.50
		**Overall**	**550**	**1.95**	**77.30**	**62.70**	**33.60**
Minimal	No weight	<50	149	2.48	67.10	48.30	28.20
		50–55	206	2.16	73.30	53.90	27.20
		55–60	151	1.69	83.40	64.90	39.70
		≥60	19	5.00	5.30	5.30	0.00
		**Overall**	**525**	**2.22**	**72.00**	**53.70**	**30.10**
Minimal	Oversample ≥60°C	<50	149	2.59	63.10	43.00	27.50
		50–55	206	2.39	70.90	50.50	25.20
		55–60	151	1.65	87.40	68.90	34.40
		≥60	19	4.01	31.60	5.30	0.00
		**Overall**	**525**	**2.29**	**72.00**	**52.00**	**27.60**

*Note*: Mean Absolute Error (MAE) across different temperature ranges for the minimal and optimal models. Results are stratified by feature set, training mode, and temperature range.

We additionally evaluated stricter quality‐control scenarios to explore the trade‐off between dataset inclusiveness and predictive accuracy. Tightening the fitting accuracy criterion to MAE ≤ 5°C (while maintaining *R* ^2^ ≥ 0.75) reduced the training set to 1933 sonications and yielded a test MAE of 1.81°C for the optimal model (2.44°C for the minimal model) (see Table S‐3). Alternatively, excluding all sonications at ≥ 60°C under the original quality‐control criteria resulted in a test MAE of 1.78°C for the optimal model (2.04°C for the minimal model) (see Table S‐3). These sensitivity analyses confirm that the main model's performance is robust and that progressively stricter selection yields incremental accuracy gains at the cost of reduced sample size. Importantly, model generalization is inherently bounded by the empirical operating space covered by the data. As illustrated in Figure 5a–b, certain regions, such as low‐power, long‐duration sonications, are sparsely sampled, leading to reduced accuracy in these regimes. Across all configurations, however, a systematic bias toward better performance in lower temperature ranges persisted (Figure 5d–e). Together, these results indicate that, while peak temperature prediction can achieve MAE values close to 2°C under well‐controlled conditions, accuracy is fundamentally constrained in sparsely explored and highly variable regimes. This emphasizes the importance of both data curation and transparent reporting of error distributions alongside mean metrics.

## 4 DISCUSSION

In this study, we analyzed a large single‐center dataset of 316 MRgFUS thermoablative procedures, including both Vim thalamotomy and STN subthalamotomy to provide a comprehensive characterization of skull‐related determinants of thermal efficiency across targets. Our results confirm the limitations of SDR as a standalone descriptor of acoustic transmission and demonstrate that a multivariate skull‐based assessment substantially improves the prediction of treatment thermal efficiency during the screening phase. Beyond patient selection, we show that these models generalize to sonication‐wise prediction of peak temperature, integrating skull properties, sonication parameters, and treatment history. Importantly, this framework enables objective, model‐driven strategies for energy titration, supporting navigation toward predefined target temperature criteria during MRgFUS procedures.

### 4.1 Target dependency of MRgFUS thermal efficiency

Our findings highlight the intrinsic target dependency of MRgFUS thermal efficiency. Despite comparable average skull metrics between groups, STN‐FUS procedures consistently exhibited lower thermal efficiency, requiring higher acoustic power and energy to reach equivalent therapeutic temperatures. This reduced efficiency may be partly explained by the significantly higher dispersion of AOI in the STN‐FUS group that together are responsible for the lower number of active transducer elements. Importantly, these results suggest that efficiency screening criteria derived from a single target (e.g., Vim) may not be directly transferable to other anatomical targets such as the STN. In this context, reliance on a single scalar metric such as SDR may be insufficient, particularly when extending treatment to deeper or more complex targets.

### 4.2 Skull properties, treatment efficiency, and patient exclusion criteria beyond SDR

MRgFUS thermal efficiency arises from the combination of multiple skull properties rather than from a single global descriptor. By jointly analyzing skull composition (SDR), geometry (ST and DT), and beam incidence (AOI), we show that these parameters are tightly interrelated and jointly modulate acoustic transmission and heating efficiency. Still, screening criteria for transcranial MRgFUS thermoablation have traditionally relied on rigid SDR‐based cutoffs, with FDA establishing a threshold of SDR > 0.4,^5^ while other centers adopt more permissive limits, often as low as 0.3.^8^ However, our results demonstrate substantial intrinsic variability in thermal efficiency even within narrow SDR intervals, particularly in the low range (SDR = 0.30–0.40). A univariate SDR‐based model explained only 48% of TER_55_ variance (see Figure S‐2), whereas incorporating additional skull descriptors through multivariate forward‐selected regression substantially improved this, a five‐feature model combining SDR/DT_avg_, DT_skw_, NoEl, AOI_avg_, and SDR/ST_skw_ achieved a cross‐validated *R* ^2^ of 0.61, with consistent generalization on an independent held‐out test set (*R* ^2^ = 0.58). Importantly, TER_55_ predictions can be directly converted to clinically actionable estimates of the acoustic energy required to reach 55°C (E_55_), with test set prediction accuracy up to *R* ^2^ = 0.48. These findings support an efficiency‐based view of acoustic transmissibility as a continuous spectrum rather than a binary eligibility criterion. Several studies have shown that low SDR primarily shifts treatments toward a lower‐efficiency regime, characterized by higher energy demands and increased technical complexity, rather than precluding clinical benefit per se.^4^, ^5^, ^6^, ^9^ In particular, Gagliardo et al., ^4^ reported that target temperatures can still be achieved in a subset of patients with low SDR values, underscoring that SDR alone does not uniquely determine achievable heating, consistent with the broad TER_55_ dispersion observed here across SDR ranges. An efficiency‐based screening approach that integrates a small number of skull descriptors to estimate expected energy demand may therefore be better suited for clinical practice than rigid SDR cutoffs alone, enabling more nuanced patient selection, realistic expectation setting, and improved procedural planning.

### 4.3 Treatment factors that influence thermal efficiency

The relationship between delivered acoustic energy and focal temperature in MRgFUS thermoablation is intrinsically non‐linear, with progressively diminishing temperature increments at higher energy and power levels (Figure 4a,b), consistent with previously reported saturation effects in transcranial focused ultrasound.^11^, ^14^ One potential contributing mechanism is the temperature‐dependent increase in acoustic absorption in bone, which may transiently alter skull transmission properties and degrade aberration correction, thereby reducing effective energy deposition at the target.^14^ Beyond skull‐related effects, sustained temperature elevations during treatment induce local tissue changes, including edema, necrosis, and alterations in perfusion and thermal conductivity. These biological processes are closely linked to thermal dose accumulation and may modify both ultrasound absorption and heat dissipation dynamics within the target region.^26^, ^27^ In this context, the inverse relationships observed between thermal efficiency, cumulative acoustic energy, and cumulative thermal dose support the hypothesis that history‐dependent physical and biological effects contribute to progressive efficiency loss during successive sonications. Together, these findings motivate the inclusion of accumulated energy and thermal dose as predictive features in temperature modeling, enabling dynamic parameter adjustment to improve thermal control. Furthermore, to maximize procedural efficiency and patient safety, these predictive physical models could be synergistically coupled with complementary real‐time clinical feedback approaches, such as quantitative intraoperative tremor monitoring using MR‐compatible accelerometers.^28^

### 4.4 Predictive thermal models in MRgFUS thermoablation

Predictive thermal modeling can support safer and more efficient MRgFUS by enabling informed, model‐guided energy titration and reducing reliance on empirical trial‐and‐error adjustments. Our results confirm that multivariate machine learning models markedly outperform traditional heuristic or single‐metric approaches, achieving a mean absolute error of 1.91°C on a fully held‐out procedure‐level test set. Prior work by Khatmullina et al.,^29^ showed that incorporating clinically accessible covariates beyond SDR, such as sonication duration, patient demographics, and thermal response to low‐temperature alignment sonications, substantially improves peak temperature prediction, reporting MAEs of ∼2.78°C for linear models and ∼1.93°C for neural networks. More recently, Xiong et al.,^30^ introduced a dedicated deep learning architecture (Fust‐Net) integrating per‐sonication parameters with patient‐specific clinical and skull metrics, achieving normalized MAEs of 1.65°C internally and 2.43°C on external datasets. Together, these findings support the importance of multivariate, patient‐aware modeling for temperature prediction, while also highlighting the challenges of generalization across centers and acquisition conditions. Within this landscape, this study extends prior findings by leveraging a substantially larger sonication‐wise dataset. Using gradient boosting models, we achieved an overall MAE of 1.91°C for the optimal feature configuration, with 60.2% of predictions within ± 2°C and 81.5% within ± 3°C. Importantly, prediction accuracy was stable and highly consistent across the clinically dominant temperature ranges (< 50°C, 50–55°C, and 55–60°C), with MAE values between 1.58 and 1.97°C. These results are comparable to those reported by Xiong et al.,^30^ whose Fust‐Net architecture involves substantially greater model complexity, while substantially improving upon the performance reported by Khatmullina et al.,^29^ despite relying on a markedly simpler model with only 6 (minimal set) to 15 (optimal set) input variables. This reduced model complexity is expected to favor robustness and generalizability across clinical settings, while maintaining competitive predictive accuracy. Notably, unlike prior studies that evaluated performance using sonication‐level random splits, our model was validated on a procedure‐level held‐out partition, providing a more conservative and clinically realistic estimate of generalization. It should be noted that data‐driven approaches, including the present work, inherently rely on statistical associations between input variables and thermal outcome, and cannot explicitly model the underlying wave propagation physics. Numerical simulation methods offer a complementary framework by modeling full three‐dimensional acoustic propagation through the skull, capturing aberration, refraction, and attenuation effects that statistical models cannot directly represent.^23^, ^31^ While such simulations require substantially greater computational resources and patient‐specific acoustic modeling, the integration of physics‐informed constraints with data‐driven prediction represents a promising direction for future work, potentially enabling not only improved temperature forecasting but also predictive control of lesion morphology.

### 4.5 Limitations

Several limitations of this study should be acknowledged. First, temperature during MRgFUS is a dynamic, spatially distributed process, whereas our models were trained to predict a single scalar outcome: the system‐reported peak average temperature around the focus coordinate. While this simplification ignores the full spatiotemporal evolution of heating and cooling, as well as off‐target thermal spread, it was chosen because current clinical routine and intraoperative decision‐making operate predominantly relying on maximum temperatures at the target. Future extensions toward spatial thermal modeling will require the integration of rapid, physics‐based ultrasound propagation models.^23^, ^32^ Moreover, MR thermometry based on proton resonance frequency shifts exhibits intrinsic noise which can be further exacerbated in certain scenarios, such as in the absence of dedicated head coils.^33^ In our dataset, off‐target thermal noise measured across treatments yielded a mean standard deviation of 2.57 ± 2.18°C (unpublished data). This thermometry variability imposes a fundamental limit on achievable prediction accuracy independent of the modeling approach. In this context, the observed MAE of 1.91°C suggests that model performance is approaching the intrinsic precision of the thermometry acquisition itself. Incorporating a thermometry noise estimate as an additional input feature could help future models account for measurement uncertainty, potentially improving prediction calibration across heterogeneous acquisition setups, such as body coil versus dedicated head coil or single‐echo versus multi‐echo thermometry sequences.^33^ Second, the dataset was retrospectively collected from a single center, which may limit generalizability across institutions, workflows, and operator strategies. We feel the large number of sonications partially mitigates this limitation. Related to this, all skull‐related measurements in our study were derived from CT scans acquired using a single vendor. While the ExAblate system compensates for multi‐vendor differences in SDR estimation, subtle vendor‐dependent variations in CT acquisition and reconstruction could affect skull‐derived metrics. Replicating and validating the proposed models at other sites using CT data from other vendors will therefore be important to assess the stability of performance across imaging platforms. Third, our findings reinforce a critical limitation of current predictive thermal models, a degraded performance at high temperatures. Sonications that achieved ≥60°C constituted only 3.3% of our dataset, and prediction error in this regime increased substantially (MAE 4.58°C; 95% prediction interval [2.2, 7.1]°C; Figure S‐5). Notably, prediction errors at ≥ 60°C were systematically positive (observed > predicted), indicating that the model consistently underestimates temperature in this range, consistent with the interpretation that these sonications represent unintended thermal overshoots driven by factors poorly captured by the available input features. Training‐fold oversampling of ≥ 60°C sonications partially mitigated this limitation, reducing MAE to 3.77°C while preserving comparable global accuracy. Nevertheless, prediction error in this regime remained substantially higher than at lower temperatures, indicating that resampling alone cannot fully compensate for sparse coverage of extreme operating conditions. It should be noted that this regime does not represent the intended therapeutic target: the vast majority of ablative sonications across centers operate within 55°C–60°C, where model accuracy was highest (MAE 1.58°C). In any case, further improvements at higher temperatures will likely require targeted data acquisition strategies, multicenter pooling, and the integration of physics‐informed constraints ^23^ to guide model behavior in sparsely sampled regimes.

Finally, while our approach was designed to be generalizable across different anatomical targets and was validated across both Vim and STN procedures, prospective validation is necessary. Future studies should assess real‐time integration into clinical workflows and evaluate whether model‐assisted parameter planning translates into improved procedural efficiency, safety, and clinical outcomes.

## 5 CONCLUSION

We show that skull‐related thermal efficiency cannot be adequately characterized by SDR alone. A multivariate description of skull properties substantially improves efficiency estimation during screening and enables accurate, sonication‐wise prediction of peak temperature when combined with sonication parameters and treatment history. These models support objective, efficiency‐informed energy titration toward predefined target temperatures, moving MRgFUS beyond static eligibility criteria toward dynamic procedural guidance. While predictive accuracy is inherently limited by data imbalance, thermometry noise, and physiological variability, continued expansion of high‐quality, multi‐center datasets and principled modeling strategies will be key to advancing predictive control and personalization in MRgFUS thermoablation.

## FUNDING INFORMATION

This work was supported by Ministerio de Ciencia e Innovación y Universidades (PID2024‐163008OB‐I00) (JAPP). MCO was supported by FPI contract (PRE2022‐103727).

## CONFLICT OF INTEREST STATEMENT

José Angel Pineda‐Pardo has received speaker honoraria from Insightec and Palex outside the submitted work. Rafael Rodríguez‐Rojas has received speaker honoraria from Insightec outside the submitted work. Jorge U. Máñez‐Miró received money from Insightec, consultant fees for moderating a session in a summit outside the submitted work. Michele Matarazzo received money from Insightec, consultant fees for moderating a session in a summit outside the submitted work. Marta Del Alamo received compensation from Abbott, Boston Scientific, and Insightec as a consultant; speaker honoraria from Palex; and reimbursement of travel expenses to attend scientific conferences from Medtronic, Boston Scientific, and Palex. Raul Martínez‐Fernández reports speaker honoraria from Insightec and Palex outside the submitted work. The other authors have no personal, financial, or institutional interest in any materials, or devices described in this article.

## ETHICAL APPROVAL

The study was approved by the Research Ethics Committee (CEIm) of HM Hospitales, Madrid, Spain (ref. 26.01.2653‐GHM). The study was conducted in accordance with the Declaration of Helsinki. Written informed consent was obtained from all patients prior to brain CT acquisition and treatment.

## Supporting information



Supporting information: mp70652‐sup‐0001‐SuppMat.docx.

## Data Availability

The data that support the findings of this study are available from the corresponding author upon reasonable request. The data are not publicly available due to privacy and ethical restrictions.
