# Supplementary material for: From Skull Density Ratio‐based eligibility to efficiency‐informed planning: Predictive modeling of thermal response in MR‐guided focused ultrasound
Source: Med Phys. 2026 Sep 10;53(9):e70652. doi: 10.1002/mp.70652 (PMC13559215; doi:10.1002/mp.70652)
Supplement: Supplementary file 1 [file MP-53-0-s001.docx]

**Table S-1. Skull profiles and derived parameters across the whole cohort and by treatment group (Vim-FUS and STN-FUS)**. Values are reported as mean (standard deviation). Group comparisons were performed using unpaired two-sample t-tests, with corresponding P-values reported. Outliers were removed from all distributions using a robust modified z-score method based on the median and median absolute deviation, with an outlier threshold of |z| > 3.5. To prevent excessive filtering, outlier removal was reverted when fewer than 80% of observations remained after filtering. Cases with DT = 0 were excluded from the analysis. Derived ratios (SDR/ST and SDR/DT) were scaled by a factor of 10 for numerical convenience.

| Skull Profile | All patients (N=316) | Vim-FUS (N=214) | STN-FUS (N=102) |  |
| --- | --- | --- | --- | --- |
| SDR_Average_ | 0.54 (0.11) | 0.53 (0.11) | 0.54 (0.10) | n.s. |
| ST_Average_ | 6.61 (1.11) | 6.64 (1.16) | 6.55 (1.01) | n.s. |
| DT_Average_ | 2.71 (0.92) | 2.74 (0.96) | 2.62 (0.80) | n.s. |
| AOI_Average_ | 13.12 (1.05) | 13.13 (0.96) | 13.09 (1.21) | n.s. |
| SDR/ST_Average_ | 0.88 (0.30) | 0.88 (0.32) | 0.88 (0.26) | n.s. |
| SDR/DT_Average_ | 2.42 (1.18) | 2.40 (1.24) | 2.45 (1.05) | n.s. |
|  |  |  |  |  |
| SDR_STDev_ | 0.15 (0.04) | 0.15 (0.04) | 0.16 (0.04) | n.s. |
| ST_STDev_ | 1.55 (0.39) | 1.54 (0.42) | 1.56 (0.34) | n.s. |
| DT_STDev_ | 1.06 (0.41) | 1.05 (0.42) | 1.07 (0.39) | n.s. |
| AOI_STDev_ | 6.02 (0.84) | 5.88 (0.79) | 6.23 (0.80) | <0.001 |
| SDR/ST_STDev_ | 0.38 (0.17) | 0.37 (0.17) | 0.40 (0.18) | n.s. |
| SDR/DT_STDev_ | 1.27 (0.51) | 1.24 (0.53) | 1.33 (0.47) | n.s. |
|  |  |  |  |  |
| SDR_Skewness_ | 0.34 (0.35) | 0.35 (0.36) | 0.32 (0.35) | n.s. |
| ST_Skewness_ | 0.19 (0.32) | 0.22 (0.34) | 0.13 (0.26) | 0.0152 |
| DT_Skewness_ | 0.58 (0.33) | 0.55 (0.33) | 0.64 (0.33) | 0.0326 |
| AOI_Skewness_ | 0.56 (0.18) | 0.54 (0.19) | 0.59 (0.16) | 0.0173 |
| SDR/ST_Skewness_ | 0.88 (0.22) | 0.86 (0.23) | 0.93 (0.17) | 0.0040 |
| SDR/DT_Skewness_ | 0.99 (0.20) | 0.98 (0.20) | 1.00 (0.18) | n.s. |
|  |  |  |  |  |
| SDR_Kurtosis_ | 0.10 (0.57) | 0.12 (0.56) | 0.06 (0.58) | n.s. |
| ST_Kurtosis_ | -0.17 (0.51) | -0.18 (0.51) | -0.14 (0.52) | n.s. |
| DT_Kurtosis_ | 0.06 (0.62) | 0.02 (0.58) | 0.16 (0.71) | n.s. |
| AOI_Kurtosis_ | 0.40 (0.39) | 0.40 (0.38) | 0.44 (0.39) | n.s. |
| SDR/ST_Kurtosis_ | 0.62 (0.34) | 0.62 (0.34) | 0.64 (0.34) | n.s. |
| SDR/DT_Kurtosis_ | 0.55 (0.36) | 0.55 (0.37) | 0.56 (0.32) | n.s. |

**Figure S-1. Temperature-energy relationship and thermal efficiency estimators.** Schematic representation of the relationship between delivered acoustic energy (E) and peak average temperature (T) for an individual treatment target. Orange dots represent individual delivered sonications, and the blue curve corresponds to a logarithmic fit of the form T = A·ln(E) + B, used to characterize the global temperature-energy response. Several complementary thermal efficiency estimators are derived from this curve. TER_max_ is defined as the temperature-to-energy ratio at the maximum achieved temperature (T_max_) and its corresponding energy (E_max_). TER_50_, TER_55_, and TER_60_ represent the temperature-to-energy ratios required to reach clinically relevant target temperatures of 50°C, 55°C, and 60°C, respectively, estimated by interpolating the fitted curve to obtain the corresponding energies (E_50_, E_55_, E_60_). TES (temperature–energy slope) corresponds to the multiplicative coefficient (A) of the logarithmic fit and reflects the overall steepness of the temperature increase per unit energy. Together, these descriptors capture complementary aspects of treatment thermal efficiency, ranging from local efficiency at specific temperature thresholds to the global sensitivity of temperature rise to energy delivery.

**Figure S-2.** Comparison of thermal efficiency prediction models using 5-fold cross-validation with procedure-level grouping (N = 178 training procedures). Left column: observed versus predicted TER₅₅ (°C/kJ). Right column: observed versus predicted E₅₅ (kJ), derived as E₅₅ = (55-37)/TER₅₅. **(A, B)** Univariate model using SDR alone. **(C, D)** Optimal multivariate model with five skull-derived features (SDR/DT_avg_, DT_skw_, NoEl, AOI_avg_, SDR/ST_skw_) selected by forward selection (adjusted R² + AIC, VIF ≤ 5). **(E, F)** Efficiency-weighted variant of the optimal model (sample weights ∝ $1/{\mathrm{TER}_{55}}^{0.8}$), designed to improve prediction accuracy for low-efficiency procedures requiring higher energy delivery. Dashed diagonals indicate perfect agreement (identity y=x).

**Figure S-3. Distribution of peak average temperatures across sonications.** Left panel shows the histogram of peak average temperatures achieved across all sonications. Dashed vertical lines indicate clinically relevant temperature thresholds at 50 °C (sub-ablative), 55 °C (ablative), and 60 °C (high-temperature regime). Right panel summarizes the proportion of sonications within each temperature range (<50 °C, 50–55 °C, 55–60 °C, and ≥60 °C), highlighting the marked imbalance toward intermediate temperature regimes and the limited representation of sonications achieving ≥60 °C.

**Table S-2.** Ten-fold cross-validation performance on the training set for peak temperature prediction (inclusion criteria: logistic fit R² ≥ 0.75, fitting MAE ≤ 7.5 °C). Folds were assigned at the sonication level, stratified by peak temperature. Results are stratified by feature set, training mode, and temperature range.

| Feature set | Training mode | Temp. range (°C) | n | MAE (°C) | ≤3 °C (%) | ≤2 °C (%) | ≤1 °C (%) |
| --- | --- | --- | --- | --- | --- | --- | --- |
| Optimal | No weight | <50 | 663 | 1.67 | 83.10 | 68.20 | 40.10 |
|  |  | 50–55 | 896 | 1.58 | 87.50 | 68.00 | 37.70 |
|  |  | 55–60 | 615 | 1.55 | 87.20 | 70.10 | 38.90 |
|  |  | ≥60 | 75 | 4.63 | 18.70 | 0.00 | 0.00 |
|  |  | Overall | 2249 | 1.70 | 83.80 | 66.30 | 37.50 |
| Optimal | Oversample ≥60 °C | <50 | 663 | 1.67 | 83.70 | 69.70 | 41.20 |
|  |  | 50–55 | 896 | 1.76 | 82.10 | 62.70 | 36.20 |
|  |  | 55–60 | 615 | 1.48 | 88.60 | 71.40 | 42.10 |
|  |  | ≥60 | 75 | 3.87 | 33.30 | 16.00 | 1.30 |
|  |  | Overall | 2249 | 1.73 | 82.70 | 65.60 | 38.10 |
| Minimal | No weight | <50 | 632 | 1.85 | 80.50 | 62.30 | 36.10 |
|  |  | 50–55 | 877 | 1.69 | 84.40 | 63.20 | 37.40 |
|  |  | 55–60 | 593 | 1.78 | 83.50 | 62.60 | 35.10 |
|  |  | ≥60 | 74 | 5.19 | 6.80 | 0.00 | 0.00 |
|  |  | Overall | 2176 | 1.88 | 80.40 | 60.60 | 35.10 |
| Minimal | Oversample ≥60 °C | <50 | 632 | 1.83 | 80.90 | 66.30 | 38.00 |
|  |  | 50–55 | 877 | 1.94 | 77.40 | 58.70 | 32.60 |
|  |  | 55–60 | 593 | 1.63 | 85.30 | 69.60 | 35.90 |
|  |  | ≥60 | 74 | 4.28 | 24.30 | 9.50 | 1.40 |
|  |  | Overall | 2176 | 1.90 | 78.80 | 62.20 | 34.00 |

Table S-3. Sensitivity analyses under stricter quality-control criteria. Tightening fitting accuracy (MAE ≤ 5°C) or excluding ≥60°C sonications yielded incremental accuracy gains at the cost of reduced sample size.

| Feature set | Training mode | Temp. range (°C) | n | MAE (°C) | ≤3 °C (%) | ≤2 °C (%) | ≤1 °C (%) |
| --- | --- | --- | --- | --- | --- | --- | --- |
| Optimal | No weight | <50 | 149 | 1.82 | 82.60 | 67.10 | 32.20 |
|  |  | 50–55 | 199 | 1.53 | 88.90 | 68.80 | 39.20 |
|  |  | 55–60 | 155 | 1.75 | 81.90 | 60.60 | 34.20 |
|  |  | ≥60 | 18 | 5.28 | 5.60 | 0.00 | 0.00 |
|  |  | Overall | 521 | 1.81 | 82.10 | 63.50 | 34.40 |
| Minimal | No weight | <50 | 149 | 2.45 | 70.50 | 51.70 | 27.50 |
|  |  | 50–55 | 199 | 2.06 | 73.90 | 56.30 | 29.60 |
|  |  | 55–60 | 155 | 2.54 | 64.50 | 38.70 | 19.40 |
|  |  | ≥60 | 18 | 5.76 | 11.10 | 5.60 | 5.60 |
|  |  | Overall | 521 | 2.44 | 67.90 | 48.00 | 25.10 |
| Optimal | No weight: Exclude ≥60 °C | <50 | 156 | 1.78 | 81.40 | 66.00 | 36.50 |
|  |  | 50–55 | 216 | 1.74 | 85.60 | 61.10 | 34.70 |
|  |  | 55–60 | 148 | 1.84 | 81.80 | 58.10 | 24.30 |
|  |  | Overall | 520 | 1.78 | 83.30 | 61.70 | 32.30 |
| Minimal | No weight: Exclude ≥60 °C | <50 | 149 | 2.39 | 66.40 | 46.30 | 24.80 |
|  |  | 50–55 | 206 | 1.88 | 83.00 | 59.20 | 30.60 |
|  |  | 55–60 | 142 | 1.90 | 76.10 | 59.90 | 33.80 |
|  |  | Overall | 497 | 2.04 | 76.10 | 55.50 | 29.80 |

**Figure S-4.** Permutation feature importance for the optimal 15-feature gradient boosting model, computed on the training set (2249 sonications). Each bar represents the mean increase in MAE (°C) after randomly permuting the corresponding feature across 20 independent repetitions; error bars indicate ±1 standard deviation. Higher values indicate greater contribution to model performance.

**Figure S-5. Calibration and prediction error distributions on the held-out test set for the optimal 15-feature gradient boosting model.** (**A, B**) Observed versus predicted peak temperature, with the dashed line indicating perfect agreement, identity line (y=x). Points are colored by temperature bin. (**C, D**) Prediction error (observed - predicted) stratified by temperature bin. Boxes indicate interquartile range, whiskers extend to 1.5× IQR, and 95% prediction intervals (2.5th-97.5th percentiles) are annotated above each bin. Left panels (**A, C**): standard training; right panels (**B, D**): with ×3 oversampling of ≥60°C sonications within training folds. Positive errors indicate model underestimation.
